# Pig genome sequence - analysis and publication strategy

**DOI:** 10.1186/1471-2164-11-438

**Published:** 2010-07-19

**Authors:** Alan L Archibald, Lars Bolund, Carol Churcher, Merete Fredholm, Martien AM Groenen, Barbara Harlizius, Kyung-Tai Lee, Denis Milan, Jane Rogers, Max F Rothschild, Hirohide Uenishi, Jun Wang, Lawrence B Schook

**Affiliations:** 1 The Roslin Institute and Royal (Dick) School of Veterinary Studies, University of Edinburgh, Roslin, UK; 2 BGI-Shenzhen, Shenzhen 518083, China; 3 Institute of Human Genetics, Aarhus University, DK-8000 Aarhus, Denmark; 4 The Wellcome Trust Sanger Institute, The Wellcome Trust Genome Campus, Hinxton, Cambridge, UK; 5 Copenhagen University, Copenhagen, Denmark; 6 Wageningen University, Animal Breeding and Genomics Centre, Wageningen, The Netherlands; 7 Institute for Pig Genetics, Beuningen, The Netherlands; 8 Korean National Institute of Animal Science, Suwon, Kyunggi-do, Korea; 9 INRA Toulouse, France; 10 The Genome Analysis Centre, Norwich, UK; 11 Department of Animal Science and Center for Integrated Animal Genomics, Iowa State University, Ames, Iowa 50011, USA; 12 National Institute of Agrobiological Sciences, Japan; 13 Department of Biology, University of Copenhagen, Copenhagen, Denmark; 14 Institute of Genomic Biology, University of Illinois, Urbana, Illinois, USA

## Abstract

**Background:**

The pig genome is being sequenced and characterised under the auspices of the Swine Genome Sequencing Consortium. The sequencing strategy followed a hybrid approach combining hierarchical shotgun sequencing of BAC clones and whole genome shotgun sequencing.

**Results:**

Assemblies of the BAC clone derived genome sequence have been annotated using the Pre-Ensembl and Ensembl automated pipelines and made accessible through the Pre-Ensembl/Ensembl browsers. The current annotated genome assembly (Sscrofa9) was released with Ensembl 56 in September 2009. A revised assembly (Sscrofa10) is under construction and will incorporate whole genome shotgun sequence (WGS) data providing > 30× genome coverage. The WGS sequence, most of which comprise short Illumina/Solexa reads, were generated from DNA from the same single Duroc sow as the source of the BAC library from which clones were preferentially selected for sequencing. In accordance with the Bermuda and Fort Lauderdale agreements and the more recent Toronto Statement the data have been released into public sequence repositories (Genbank/EMBL, NCBI/Ensembl trace repositories) in a timely manner and in advance of publication.

**Conclusions:**

In this marker paper, the Swine Genome Sequencing Consortium (SGSC) sets outs its plans for analysis of the pig genome sequence, for the application and publication of the results.

## Background

The pig genome is being sequenced and characterised under the auspices of the Swine Genome Sequencing Consortium [1]. A Data Release Workshop convened in Toronto in May 2009 by Genome Canada and other funding agencies affirmed and extended the commitments to prepublication release of large data sets in the life sciences which were originally developed in the context of the Human Genome Project. The Toronto Statement [2] places obligations on the producers of such data sets, including genome sequence data, in respect of prepublication release of the data and confirms the principle that allows the data producers to publish the first global analyses of the data set. The data producers are encouraged to produce a citable statement or "marker paper" in which they describe the data set and their intentions in respect of analysis and publication. In this marker paper, the Swine Genome Sequencing Consortium (SGSC) sets outs its plans for analysis of the pig genome sequence, for the application and publication of the results. These plans were presented to participants in the Pig Genome III conference held at the Wellcome Trust Sanger Institute, 2-4 November 2009.

## Results

### Pig genome sequence data

The sequence data from which a draft pig genome sequence will be assembled comprises hierarchical shotgun sequence data providing 4-6× genome coverage from BAC clones representing a minimal tile path across the genome plus > 30× genome coverage in whole genome shotgun sequence (WGS) data generated using Sanger (capillary) and next-gen (Illumina) technologies. The minimal tile path was identified from a high quality physical (BAC contig) map [3] and provides coverage of 98.3% of this physical map. As at 5^th ^July 2010 the total length of the BAC-derived sequence contigs, prior to the removal of sequence redundancy between overlapping BAC clones, was 3.01 Gbp of which 156.3 Mbp was at finished quality. These sequence data were generated from 16,707 BAC clones of which 15,895 have been subjected to one round of automated pre-finishing.

### Prepublication data release

In accordance with the Bermuda and Fort Lauderdale agreements and the more recent Toronto Statement [2] the data have been released into public sequence repositories (Genbank/EMBL, NCBI/Ensembl trace repositories) in a timely manner and in advance of publication. Assemblies of the genome sequence have been annotated using the Pre-Ensembl and Ensembl automated pipelines and made accessible through the Pre-Ensembl/Ensembl browsers. The current annotated genome assembly (Sscrofa9) was released with Ensembl 56 in September 2009. The current assembly (Sscrofa9) was constructed entirely from the BAC-derived sequence data.

### Analysis strategy

A revised assembly (Sscrofa10) is being constructed from the BAC clone derived sequence together with the WGS data. The publication of a draft genome sequence for the pig will be based on this new assembly. A series of analysis working groups have been established in consultation with the pig genome research community under the auspices of the SGSC in order to undertake genome-wide analyses of the genome sequence. These groups with their respective lead contacts are summarised in Table 1. Details of the work of these groups will be posted on the SGSC website at http://www.piggenome.org.

**Table 1:** Swine Genome Sequencing Consortium genome sequence analysis groups

Analysis group	Lead contact	Notes
Assembly	Alan Archibald alan.archibald@roslin.ed.ac.uk	The target for the next assembly is to incorporate all the available sequence data for Duroc 2-14, including BAC clones sequences, WGS Sanger and next-generation short sequence reads. Contig and scaffold order and orientation will be tested against other genome maps and in particular the high resolution radiation hybrid maps.

Structural variation, segmental duplication, copy number variation	Christian Bendixen christian.bendixen@agrsci.dk	The reference genome sequence will be analysed for evidence of segmental duplications. Comparative Genomic Hybridisation data, paired-end and mate-pair re-sequence data from other pigs will be used to identify smtructural and copy number variation.

Repetitive DNA, transposable elements Speciation, wild and related suids and selection	Geoff Faulkner geoff.faulkner@roslin.ed.ac.uk Lawrence Schook schook@uiuc.edu	Retroviruses and related repetitive sequences in Sus scrofa and related species will be characterized. Sequence and 60 K SNP genotype data from wild boar and related species will be explored to address the origins of domestic pigs. Comparative sequence analyses of domesticated and wild boar genome sequences is expected to reveal signatures of artificial and natural selection.

Evolution	Leif Andersson Leif.andersson@imbim.uu.se	Natural and artificial selection will have shaped the pig genome sequence. Comparison of the pig genome sequence with the sequences of other mammals is expected to reveal genes that are evolving more rapidly in the pig and artiodactyl lineages.

Comparative genomics	Martien Groenen Martien.groenen@wur.nl	Genome rearrangements and conserved synteny compared to other suids and other mammals.

Imprinting	Ole Madsen Ole.madsen@wur.nl	RNA-seq data from a range of tissues from Duroc 2-14 or her clones will be analysed to identify genes that show differential allelic expression and potentially imprinted genes.

SNP	Martien Groenen martien.groenen@wur.nl	Re-sequence data and the WGS sequence data from Duroc 2-14 will be examined for putative SNPs and small indels, including those for which Duroc 2-14 is heterozygous.

ncRNA	Jan Gorodkin gorodkin@genome.ku.dk	The genome sequence will be explored for putative ncRNA sequences and microRNA encoding loci.

Gene builds	Steve Searle Searle@sanger.ac.uk	The Ensembl automated pipeline will be used to establish a Gene Build for the pig genome that will be compared with builds generated by other systems including NCBI.

Protein interactions	Soren Brunak brunak@cbs.dtu.dk	Development of a proteome will be initiated.

Immune genes	Chris Tuggle cktuggle@iastate.edu	The immune gene analysis group will manually annotate pig genes predicted/known to have roles in the immune system. The repertoire of pig immune genes will be examined for evidence of pig-lineage specific features.

Reproduction	Max Rothschild mfrothsc@iastate.edu	The reproduction gene analysis group will manually annotate pig genes predicted/known to have roles in reproductive functions and seek to identify pig-lineage specific features.

Obesity	Max Rothschild mfrothsc@iastate.edu	The obesity gene analysis group will manually annotate pig genes predicted/known to have roles in obesity and seek to identify pig-lineage specific features

Olfaction, neuropeptide and prohormone	Sandra Rodriguez-Zas rodrgzzs@illinois.edu	Approximately 5% of the genes in the Sscrofa9 Gene Build are predicted to have olfactory functions. These genes will be manually annotated and examined for pig-specific characteristics. In addition, the neuropeptide and prohormone gene families will be annotated.

Manual annotation	Jim Reecy jreecy@iastate.edu	The pig research community is engaged in efforts to manually Annotate genes identified/predicted by the Ensembl analysis pipeline. The otterlace system will be used to enable this community annotation activity.

Biomedical Models	Lawrence Schook schook@illinois.edu	The use of genomic information to enhance the utilization of the pig in xenotransplantation and as a model for cardiovascular, cancer and obesity will be addressed. How genomic information supports the further development of transgenic pigs for creating essential animal models will also be discussed.

### Publication strategy

The Swine Genome Sequencing [1] and Swine HAPMAP [4] consortia respectively propose to develop two summary papers for publication describing a) the sequencing and analysis of the pig genome and b) genetic variation and haplotype structures across a range of pig breeds and related Sus species. In addition, the consortia propose to develop a series of companion papers describing either the results from the analysis groups and/or results from other research projects that have been enabled by the publication of a draft sequence of the pig genome. The consortia would be pleased to hear from research groups with plans for manuscripts that could be included within the list of companion papers. Please address correspondence to either Alan Archibald alan.archibald@roslin.ed.ac.uk or Larry Schook schook@illinois.edu.

## Discussion

The value of the pig genome sequence lies not only in shaping the continued use of pigs in agriculture and medical research but also in the realm of evolution and domestication (natural and artificial selection) [5]. The pig is an economically important species not only as a major source of meat-based protein but also increasingly as a model for biomedical research. For example, the pig has value as a model of a spectrum of human diseases that may be modelled less well in rodents, including obesity, arthritis and cardiovascular disease.

The domestic pig (*Sus scrofa*) is a eutherian mammal and a member of the Cetartiodactyla order, a clade distinct from rodent and primates that last shared a common ancestor with humans between 79 and 87 million years ago. The domestic pig belongs to the Suidea family that consists of multiple species, all found in Asia, Europe and Africa. The availability of this wide variety of pig species that diverged over a period of around 2 to 15 million years provides a rich resource to study genomic changes in relation to speciation. A well characterised pig genome sequence forms a template for the study of within and between species genetic variation. Our analysis of the pig genome sequence will be set in the context of parallel research on the genomes of closely related and contemporary Suids (e.g. *Sus verrocus*, *Sus celebensis and Sus barbatus*) and on within breed genetic variation using the 60 K pig SNP chip [4] and by re-sequencing.

## Conclusions

The pig genome sequencing project has been conducted in an open international collaborative manner in the spirit of the Bermuda and Fort Lauderdale agreements. In accordance with the more recent Toronto Statement the sequence data have been released in advance of publication. In this marker paper, the Swine Genome Sequencing Consortium (SGSC) sets outs its plans for analysis of the pig genome sequence, for the application and publication of the results.

## Methods

### Sequencing strategy

**Figure 1:**
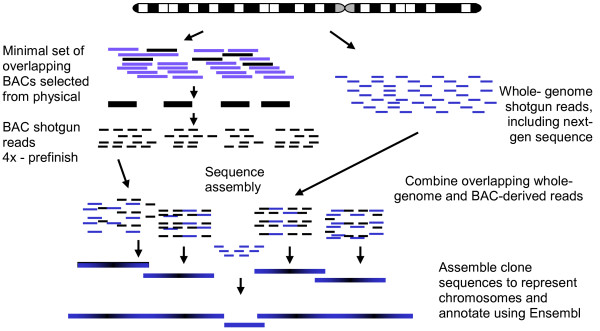
**Sequencing strategy - a hybrid approach combining hierarchical shotgun sequencing of BAC clones and whole genome shotgun sequencing**.

The pig genome has been sequenced following a hybrid approach representing a refinement of the strategy announced earlier [1] (Figure 1). Briefly, BAC clones selected to represent a minimal tile path across the genome were identified from the high resolution physical (BAC contig) map [2] and were subjected to hierarchical shotgun sequencing. BAC clones from the CHORI-242 library prepared from DNA from a single Duroc sow (Duroc 2-14) were preferentially chosen for sequencing. The initial plan was to skim sequence the BAC clones to 3× coverage. In practice, both ends of 768 subclones for each BAC were sequenced (average read length of 707 bp) to provide ~4× coverage. Most BAC clones have subsequently been subjected to one round of automated pre-finishing by primer walking from the ends of the clone sequence contigs constructed from the initial 4× coverage skim sequencing. This hierarchical shotgun sequencing was primarily undertaken at the Wellcome Trust Sanger Institute, with additional clones sequenced by the National Institute of Agrobiological Sciences, Japan. In addition whole genome shotgun (WGS) sequence data were generated from DNA isolated from the same animal (Duroc 2-14). These WGS data were generated using both Sanger capillary sequencing at the Korean Livestock Research Institute and Illumina/Solexa sequencing at the Beijing Genomics Institute and the Wellcome Trust Sanger Institute.

## Authors' contributions

All authors are members of the Swine Genome Sequencing Consortium (SGSC) under whose auspices the pig genome is being sequenced. They are responsible for securing the funding for, and the management of, the pig genome sequencing project. ALA, DM, JR, MFR and LBS are members of the SGSC Steering Committee. ALA, MF, DM, JR, MFR, HU and LBS are members of the SGSC Technical Committee. LBS, CC, ALA, MAMG, DM, JR, MF, MFR comprise the SGSC Manuscript Steering Committee which is directing the SGSC's analysis and publication strategy. JR and CC led the sequencing team at the Wellcome Trust Sanger Institute which generated the BAC clone derived sequence data, during the initial and later stages of the project, respectively. LBS and JR were co-directors of the USDA grant which provided ca. 50% of the project funding. MAMG was work package leader for the EC-funded project to sequence chromosomes 7 and 14. BH and MAMG were project leaders for the IPG-funded project to sequence chromosome 4. ALA was the PI for the BBSRC grant on annotation and analysis. MFR secured US pig industry funding for the project and led a pilot project to generate finished sequence for part of chromosome 17. JW led the Beijing Genomics Institute effort to generate WGS data using Illumina next-gen sequencing technology partially funded by a grant of which LB was the PI. K-TL led the team at the Korean Livestock Research Institute that has contributed WGS data using Sanger capillary technology. HU leads the team at Japanese National Institute of Agrobiological Sciences which contributed full length cDNA sequence and some BAC clone sequence data. DM leads the team which is validating the sequence assembly against a high resolution radiation hybrid map. Finally, some of the leadership roles of the authors in the analysis of the sequence data are highlighted in Table 1. All authors have read and approved the manuscript.
